# OPTINOFA – Intelligenter Assistenzdienst zur strukturierten Ersteinschätzung in der Notaufnahme

**DOI:** 10.1007/s00063-024-01126-y

**Published:** 2024-03-27

**Authors:** Elisabeth Nyoungui, Marina V. Karg, Marc Wieckenberg, Katrin Esslinger, Michael Schmucker, Andreas Reiswich, Kai L. Antweiler, Tim Friede, Martin Haag, Harald Dormann, Sabine Blaschke

**Affiliations:** 1 Zentrale Notaufnahme, Universitätsmedizin Göttingen, Robert-Koch-Str. 40, 37075 Göttingen, Deutschland; 2 Zentrale Notaufnahme, Klinikum Fürth, Fürth, Deutschland; 3 Zentrale Notaufnahme, Evangelisches Krankenhaus Göttingen-Weende, Göttingen, Deutschland; 4 Institut für Medizinische Informatik, Universitätsmedizin Göttingen, Göttingen, Deutschland; 5 GECKO Institut, Hochschule Heilbronn, Heilbronn, Deutschland; 6 Institut für Medizinische Statistik, Universitätsmedizin Göttingen, Göttingen, Deutschland

**Keywords:** Notfallmedizin, Triage, Algorithmus, Organisatorische Entscheidungsfindung, Entscheidungsunterstützende Techniken, Emergency medicine, Triage, Algorithm, Organizational decision-making, Decision support techniques

## Abstract

**Hintergrund:**

Seit Beginn der letzten Dekade ist in Deutschland ein Anstieg der Fallzahlen in den Zentralen Notaufnahmen (ZNA) der Krankenhäuser zu verzeichnen, der zu rezidivierenden Overcrowding-Szenarien sowie zur Erhöhung der Risiken und Kosten der Notfallbehandlung führt. Dabei hat der Anteil ambulanter Notfallbehandlungen überproportional zugenommen. Im Rahmen des Innovationsfondsprojekts Optimierung der Notfallversorgung durch strukturierte Ersteinschätzung mittels intelligenter Assistenzdienste (OPTINOFA, Förderkennzeichen [FKZ] 01NVF17035) wurde hierzu ein intelligenter Assistenzdienst entwickelt.

**Patient:innen und Methodik:**

Auf Basis etablierter Triagesysteme (Emergency Severity Index, ESI; Manchester Triage System, MTS) wurde für die 20 häufigsten Leitsymptome ein neuer Triagealgorithmus entwickelt und webbasiert auf mobilen Endgeräten zur Verfügung gestellt. Zur Bewertung der Validität, Reliabilität und Sicherheit des neuen Triageinstruments OPTINOFA wurde nach positivem Ethikvotum eine Pilotstudie in 3 ZNAs durchgeführt.

**Ergebnisse:**

In die Pilotstudie wurden *n* = 718 Notfallpatienten (59 ± 22 Jahre; 349 männlich, 369 weiblich) eingeschlossen. In Bezug auf die Disposition (ambulant/stationär) ergab sich mit OPTINOFA eine Sensitivität von 91,1 % bei einer Spezifität von 40,7 % sowie eine gute Korrelation zur OPTINOFA-Triagestufe (Spearman’s Rank Correlation ρ = 0,41). In Bezug auf die Prädiktion der Disposition gemäß OPTINOFA-Triagestufe lag die „area under the curve“ (AUC) bei 0,73. Das intrahospitale Überleben von Patient:innen mit der OPTINOFA-Triage-Stufe 4 bzw. 5 lag bei 100 %. Die Assoziation zwischen der Verweildauer in der Notaufnahme und der OPTINOFA-Triage-Stufe war signifikant (*p* < 0,0001).

**Schlussfolgerungen:**

Die Pilotstudie zeigt, dass OPTINOFA ein sicheres und valides Triagesystem zur transsektoralen Steuerung der Patientenströme in der Notaufnahme ist. Durch Festlegung von Behandlungsdringlichkeit und Versorgungssektor ergeben sich hiermit bedarfsgerechte Perspektiven zur Entlastung der ZNA durch engere Verzahnung zwischen den Sektoren der Notfallversorgung.

**Zusatzmaterial online:**

Zusätzliche Informationen sind in der Online-Version dieses Artikels (10.1007/s00063-024-01126-y) enthalten.

## Abstract (en)

**Background:**

Case numbers in central emergency departments (EDs) have risen during the past decade in Germany, leading to recurrent overcrowding, increased risks in emergency care, and elevated costs. Particularly the fraction of outpatient emergency treatments has increased disproportionately. Within the framework of the Optimization of emergency care by structured triage with intelligent assistant service (OPTINOFA, Förderkennzeichen [FKZ] 01NVF17035) project, an intelligent assistance service was developed.

**Patients and methods:**

New triage algorithms were developed for the 20 most frequent leading symptoms on the basis of established triage systems (emergency severity index, ESI; Manchester triage system, MTS) and provided as web-based intelligent assistance services on mobile devices. To evaluate the validity, reliability, and safety of the new OPTINOFA triage instrument, a pilot study was conducted in three EDs after ethics committee approval.

**Results:**

In the pilot study, *n* = 718 ED patients were included (age 59.1 ± 22 years; 349 male, 369 female). With respect to disposition (out-/inpatient), a sensitivity of 91.1% and a specificity of 40.7%, and a good correlation with the OPTINOFA triage levels were detected (Spearman’s rank correlation ρ = 0.41). Furthermore, the area under the curve (AUC) for prediction of disposition according to the OPTINOFA triage level was 0.73. The in-hospital mortality rate of OPTINOFA triage levels 4 and 5 was 0%. The association between the length of ED stay and the OPTINOFA triage level was shown to be significant (*p* < 0.001).

**Conclusion:**

The results of the pilot study demonstrate the safety and validity of the new triage system OPTINOFA. By definition of both urgency and emergency care level, new customized perspectives for load reduction in German EDs via a closer cooperation between out- and inpatient sectors of emergency care could be established.

Der Status quo der Notfallversorgung in Deutschland ist gemäß Gutachten des Sachverständigenrats für Gesundheit (2018) derzeit nicht bedarfsgerecht: So kommt es insbesondere durch die fehlende Steuerung der Patientenströme zu einer Über‑, Unter- oder Fehlversorgung der Notfallpatient:innen in den zentralen Notaufnahmen (ZNA) der Krankenhäuser. Im Rahmen des Innovationsfondsprojekts Optimierung der Notfallversorgung durch strukturierte Ersteinschätzung mittels intelligenter Assistenzdienste (OPTINOFA, Förderkennzeichen [FKZ] 01NVF17035) wurde dazu ein neues System zur strukturierten Ersteinschätzung von Notfällen mit Festlegung von Behandlungsdringlichkeit und erforderlichem Versorgungssektor entwickelt, um dieses Problem zu adressieren. Die Anwendung des neuen Triageinstruments OPTINOFA wurde in einer Pilotstudie in drei ZNA validiert.

## Hintergrund

In Deutschland verzeichneten die Zentralen Notaufnahmen (ZNA) der Krankenhäuser seit Beginn der letzten Dekade einen Fallzahlanstieg von 24,9 Mio. im Jahr 2009 auf 27,8 Mio. im Jahr 2019 [19]. Seit dem Jahr 2019 kam es zu einem Rückgang auf 22,6 Mio. im Jahr 2021 und seitdem zu einer Plateaubildung [28]. Neben der hohen Patientenfrequentierung in den ZNA kommt es aufgrund von Personalengpässen, Mangel an stationären Versorgungskapazitäten und Patientenzuweisungen trotz Kapazitätserschöpfung zu massiven Belastungssituationen [18, 20]. In der Folge treten Overcrowding-Szenarien auf [25], die zu Verzögerungen in der Notfallversorgung, langen Wartezeiten sowie Verweildauern und damit einem Anstieg der Risiken der Notfallversorgung bis hin zu einer Erhöhung der Mortalitätsrate führen [3].

Neben der ärztlichen und Rettungsdienstzuweisung definiert sich der Selbsteinweisende in der ZNA aus seiner subjektiven Wahrnehmung als Notfallpatient:in [22]. In der Konsequenz werden hierdurch ambulante und stationäre Fälle nach ärztlicher Behandlung in den ZNA generiert [1]. Die Art der Zuweisung zu einer ZNA, also mit oder ohne Rettungsmittel, als Selbsteinweiser oder über hausärztliche Einweisung, lässt keine Prognose über die benötigten Ressourcen oder den Status ambulant oder stationär zu [2, 13]. Andererseits obliegt gemäß § 75 Sozialgesetzbuch (SGB) V die Sicherstellung der ambulanten Notfallversorgung außerhalb der Praxisöffnungszeiten der kassenärztlichen Vereinigung (KV). Der/die Notfallpatient:in hat aber unabhängig davon gemäß § 76 SGB V Satz 2 das Recht auf freie Arztwahl im Notfall.

Im Rahmen der gestuften Notfallversorgung wird gemäß Richtlinie des Gemeinsamen Bundesausschusses (G-BA) eine medizinische Erstsichtung zur Einschätzung der Behandlungsdringlichkeit (sog. Triage) innerhalb von 10 min gefordert [8]. Diese wird zwar bereits bundesweit durch etablierte Triagesysteme, wie z. B. Emergency Severity Index (ESI) oder Manchester Triage System (MTS; [6]), umgesetzt; eine Berücksichtigung der Versorgungsebene (ambulant vs. stationär) wurde bisher in den validierten Triagealgorithmen jedoch nicht evaluiert. Eine effiziente Nutzung des Gesundheitssystems im Notfall setzt aber voraus, dass der richtige Patient am richtigen Ort zur richtigen Zeit diagnostiziert und behandelt wird. In der aktuellen Gesetzgebung wird daher der Fokus auf eine optimale Patientensteuerung für Notfallpatient:innen gefordert. Im § 120 Absatz 3b Gesundheitsversorgungsweiterentwicklungsgesetz (GVWG) wurde der G‑BA aufgefordert, Richtlinien zur „Ersteinschätzung des medizinischen Versorgungsbedarfs von Hilfesuchenden, die sich zur Behandlung eines Notfalls an ein Krankenhaus wenden“ zu erarbeiten. Konkret werden die Anforderungen an das standardisierte Ersteinschätzungsinstrument definiert, das unter anderem sowohl die Behandlungsdringlichkeit als auch eine Empfehlung für die geeignete Versorgungsebene und dessen digitale Dokumentation bereitstellen soll [9].

Da es bis dato kein validiertes System für die Steuerung der Patientenströme im Bereich der medizinischen Notfallversorgung in Deutschland gibt, wurde im Rahmen des vom Innovationsfonds geförderten Projekts OPTINOFA ein neuer intelligenter und webbasierter Assistenzdienst entwickelt, der im Rahmen einer Notaufnahmevorstellung eine strukturierte Ersteinschätzung der Behandlungsdringlichkeit und Versorgungsstufe von Notfallpatient:innen und damit eine sektorenspezifische Zuweisung ermöglicht. Zur Validierung des Assistenzdiensts wurde eine Pilotstudie in drei ZNA durchgeführt. Die Qualität des Triageinstruments wurde in Bezug auf Validität, Reliabilität sowie Korrelation der OPTINOFA-Triage-Stufen mit Disposition, Mortalität sowie Krankenhaus- und Notaufnahmeverweildauer als Gütekriterien bewertet.

## Patient:innen und Methoden

### Entwicklung des Assistenzdiensts OPTINOFA

Im Rahmen des Projekts OPTINOFA wurden auf Basis der Standardarbeitsanweisungen (SOP) im „SOP Handbuch Interdisziplinäre Notaufnahme“ [4], etablierter Triagesysteme [6], der aktuellen Leitlinien, einer systematischen Literaturrecherche sowie des Kriterienkatalogs für die sektorenspezifische Zuweisung der Notfallpatient:innen neue Notfallalgorithmen für die strukturierte Ersteinschätzung (Triage) entwickelt. Hierbei war es Ziel, für die 20 häufigsten, notfallmedizinischen Leitsymptome (Tab. 1; [12]) gemäß dem Code des Canadian Emergency Department Information System (CEDIS, V3.0; [5]) eine strukturierte Ersteinschätzung der Behandlungsdringlichkeit und der erforderlichen Notfallversorgungsstufe zu definieren.

**Tab. 1:** Die 20 häufigsten notfallmedizinischen Leitsymptome [12]

Vorstellungsgrund	Relative Häufigkeit (%)	CEDIS-Code
Schmerzen obere Extremität	10,9	554
Schmerzen untere Extremität	10,9	555
Bauchschmerzen	7,0	251
Verletzung obere Extremität	5,4	556
Rückenschmerzen	4,0	551
Allgemeine Schwäche	4,0	007
Luftnot	3,8	651
Brustschmerz (kardial)	3,7	003
Hypertonie	2,6	006
Schwäche in den Extremitäten/Symptome eines Schlaganfalls	2,4	409
Verletzung untere Extremität	2,2	557
Kopfverletzung	1,9	407
Schwindel	1,7	403
Kopfschmerz	1,6	404
Brustschmerz (nichtkardial)	1,5	004
Palpitationen/unregelmäßiger Herzschlag	1,4	005
Flankenschmerz	1,2	301
Übelkeit und/oder Erbrechen	1,1	257
Harnverhalt	1,1	306
Ohrenschmerzen	0,9	051

*CEDIS* Canadian Emergency Department Information System

**Abb. 1:**
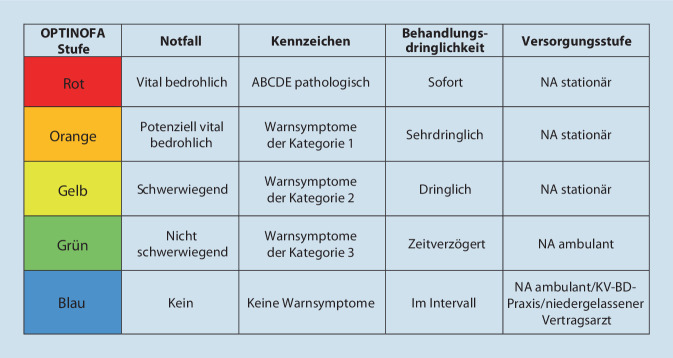
OPTINOFA-Triage-Stufen. *ABCD* *A* Airway, *B* Breathing, *C* Circulation, *D* Disability, *E* Exposure/Environment, *NA ambulant* ambulante Versorgung in der Notaufnahme, *NA stationär* stationäre Versorgung in der Notaufnahme, *KV-BD-Praxis* Bereitschaftsdienstpraxis der Kassenärztlichen Vereinigung

Für jedes Leitsymptom erfolgte im Rahmen eines Konsensverfahrens eine Festlegung und Wertung von definierten Warnsymptomen mit dazugehöriger Kategorisierung in Bezug auf Behandlungsdringlichkeit und Versorgungsstufe. Dies erfolgte in einem Expertengremium bestehend aus je 2 fachärztlichen Expert:innen der Fachdisziplinen klinische Notfall- und Akutmedizin sowie Allgemeinmedizin. Die Qualitätssicherung des Verfahrens wurde über den wissenschaftlichen Beirat des Projekts sichergestellt. Die Notfallalgorithmen wurden spezifisch für jedes Leitsymptom in Form eines Flussdiagramms konzipiert und ein 5‑stufiges System für die strukturierte Ersteinschätzung von Behandlungsdringlichkeit und Versorgungsstufe festgelegt (Abb. 1).

### Pilotstudie

Zur Validierung des intelligenten Assistenzdiensts OPTINOFA wurde schließlich in den Notaufnahmen von drei Modellkliniken auf Basis des positiven Votums der federführenden Ethikkommission der Universitätsmedizin Göttingen (UMG) (Antragsnummer 20/12/18) sowie eines umfassenden Datenschutzkonzepts eine Pilotstudie durchgeführt. Die drei Modellkliniken umfassten die ZNA in der UMG (Notfallstufe 3, 50–60.000 Fälle pro Jahr), das Evangelische Krankenhaus Göttingen-Weende (EKW, Notfallstufe 2, 25–30.000 Fälle pro Jahr) und das Klinikum Fürth (KFÜ, Notfallstufe 3, 50.000 Fälle pro Jahr). Hierbei erfolgte über einen Zeitraum von 4 Wochen im EKW und 8 Wochen im UMG und im KFÜ jeweils parallel eine Triage nach ESI (UMG, KFÜ) bzw. MTS (EKW) und OPTINOFA. Die strukturierte Ersteinschätzung wurde hierbei jeweils in allen drei Modellkliniken durch qualifizierte Triage Nurses durchgeführt, die speziell für OPTINOFA geschult wurden. Die Rekrutierung erfolgte werktags im Früh- und/oder Spätdienst zu Öffnungszeiten der Bereitschaftsdienstpraxen.

Die Einschlusskriterien umfassten ein Alter ≥ 18 Jahre sowie das Vorhandensein eines der 20 häufigsten Leitsymptome (Tab. 1; [12]). Notfallpatient:innen unter 18 Jahre und solche ohne die genannten Leitsymptome wurden ausgeschlossen. Darüber hinaus wurden keine Notfälle mit einer „Do-not-resuscitate“-Order oder einer Vorsorgevollmacht in die Studie eingeschlossen. Dokumentiert wurden der CEDIS-Code der Leitsymptome/Vorstellungsgrund, die ESI- bzw. MTS-Triage-Stufe (1 = rot, 2 = orange, 3 = gelb, 4 = grün, 5 = blau), die OPTINOFA-Triage-Stufe (1 = rot, Notaufnahme stationär; 2 = orange, Notaufnahme stationär; 3 = gelb, Notaufnahme stationär; 4 = grün, Notaufnahme ambulant; 5 = blau, KV-Bereitschaftsdienstpraxis oder niedergelassener Arzt) sowie die Zeitdauer der Ersteinschätzung. Darüber hinaus wurden aus der Datenbank des Krankenhausinformationssystems die Aufnahme‑, Entlassungs- bzw. Verlegungsdiagnose, die Zielstation bei stationärer Aufnahme, Alter und Geschlecht, die Notaufnahmeverweildauer (VWD), die Krankenhaus-VWD und ggf. die Krankenhausmortalität (Tod während des Krankenhausaufenthalts) erfasst. Zur Beurteilung der Dringlichkeit wurde retrospektiv der klinische Verlauf bewertet: Das Kriterium „hohe Dringlichkeit“ war erfüllt, wenn eine Behandlung auf der Intensivstation (ICU), Intermediate-Care (IMC)-Station, auf der Stroke Unit oder im Schockraum stattgefunden hatte, eine sofortige Operationsindikation gestellt wurde oder der Patient verstarb.

### Statistik

Für den Zusammenhang zwischen Triagestufe und Disposition (Ort der Behandlung) wurden Spearman-Korrelationen berechnet. Die vier Orte wurden hierbei in der Reihenfolge Ambulanz, Normal‑, IMC- und ICU angeordnet. Die Analyse wurde nach einer Zusammenfassung der drei stationären Orte wiederholt, sodass gezielt die beiden Versorgungsbereiche Ambulanz und Station untersucht wurden. Für diese wurde anschließend eine Receiver-operating-characteristic(ROC)-Analyse durchgeführt und ein *p*-Wert mit dem Verfahren von De Long ermittelt. Die Abhängigkeit zwischen Triagestufen und Zeitmessungen in Minuten wurden mittels Analyse der Varianz (ANOVA) getestet; die Abhängigkeit zwischen den Triagestufen und Zeitmessungen in Tagen mittels Kruskal-Wallis-Tests. Tod während des Krankenhausaufenthalts wurde mittels Kaplan-Meier-Kurven dargestellt. Entlassungen gingen hierbei als Zensierungen in die Berechnungen ein. Stetige Größen wurden als Mittelwert mit Standardabweichung angegeben. Für Zeitdauern wurde der Median mitaufgeführt. Absolute und relative Häufigkeiten wurden für kategoriale Variablen dargestellt. Testergebnisse mit einem *p*-Wert von höchstens 0,05 wurden als signifikant bezeichnet. Eine Adjustierung bezüglich der Anzahl der durchgeführten Tests wurde nicht vorgenommen. Für statistische Analysen wurde SAS® 9.4 (SAS, Cary, NC, USA) verwendet.

## Ergebnisse

### Charakteristika des Patientenkollektivs in der Pilotstudie

Innerhalb des Beobachtungszeitraums wurden in den drei Modellkliniken insgesamt *n* = 718 Patient:innen, davon 51,4 % weiblich, mit einem mittleren Alter von 59,1 Lebensjahren (Standardabweichung; STD = ±22 Jahre) eingeschlossen. In der UMG wurden dabei *n* = 288 Patient:innen rekrutiert, davon 48,4 % weiblich, *n* = 240 im KFÜ, davon 58,3 % weiblich, und *n* = 190 im EKW, davon 47,4 % weiblich. Die Ergebnisse der deskriptiven Statistik sind in Tab. 2 kumuliert für das gesamte Patient:innenkollektiv sowie differenziert nach Modellklinik dargestellt (Tab. 2).

**Tab. 2:** Charakteristika der Notfallpatient:innen in den Modellkliniken Universitätsmedizin Göttingen (*UMG)*, Klinikum Fürth (*KFÜ*) und Evangelisches Krankenhaus Göttingen-Weende (*EKW*) sowie Ergebnisse der Pilotstudie

Charakteristika	UMG	KFÜ	EKW	Gesamt
	*n* = 288	*n* = 240	*n* = 190	*n* = 718
*Demografische Daten*				
*Alter, M (STD)*	58,2 (21,5)	59,9 (22,4)	59,3 (22,5)	59,1 (22,0)
*Geschlecht*				
männlich	149 (51,7 %)	100 (41,7 %)	100 (52,6 %)	349 (48,6 %)
weiblich	139 (48,3 %)	140 (58,3 %)	90 (47,4 %)	369 (51,4 %)
*Leitsymptome (CEDIS-Codes)*				
*Kardiovaskulär (001–050)*	93 (50,8 %)	53 (29 %)	37 (20,2 %)	183 (25,5)
003 Brustschmerz kardial	28 (9,7 %)	17 (7,1 %)	7 (3,7 %)	52 (7,2 %)
004 Brustschmerz nichtkardial	6 (2,1 %)	2 (0,8 %)	2 (1,1 %)	10 (1,4 %)
005 Palpitationen	13 (4,5 %)	15 (6,2 %)	4 (2,1 %)	32 (4,5 %)
006 Hypertonie	8 (2,8 %)	6 (2,5 %)	0 (0 %)	14 (1,9 %)
007 Allgemeine Schwäche	38 (13,2 %)	13 (5,4 %)	24 (12,6 %)	75 (10,4 %)
*HNO (Ohren; 051–100)*	1 (0,3 %)	3 (1,7 %)	0 (0 %)	4 (0,5 %)
051 Ohrenschmerzen	1 (0,3 %)	3 (1,7 %)	0 (0 %)	4 (0,5 %)
*Gastrointestinal (251–300)*	58 (46,8 %)	37 (29,8 %)	29 (23,4 %)	124 (17,3 %)
251 Bauchschmerzen	46 (16 %)	30 (12,4 %)	25 (13,2 %)	101 (14 %)
257 Übelkeit/Erbrechen	12 (4,2 %)	7 (2,9 %)	4 (2,1 %)	23 (3,2 %)
*Urogenital (301–350)*	2 (18,2 %)	9 (81,8 %)	0 (0 %)	11 (1,5 %)
301 Flankenschmerzen	2 (0,7 %)	6 (2,5 %)	0 (0 %)	8 (1,1 %)
306 Harnverhalt	0 (0 %)	3 (1,2 %)	0 (0 %)	3 (0,4 %)
*Neurologisch (401–450)*	92 (63,5 %)	45 (31 %)	8 (5,5 %)	145 (20,2 %)
403 Schwindel	20 (6,9 %)	8 (3,3 %)	0 (0 %)	28 (3,9 %)
404 Kopfschmerzen	21 (7,3 %)	13 (5,4 %)	0 (0 %)	34 (4,7 %)
407 Kopfverletzung	1 (0,3 %)	12 (5 %)	7 (3,7 %)	20 (2,8 %)
409 Schwäche der Extremitäten	50 (17,4 %)	12 (5 %)	1 (0,5 %)	63 (8,8 %)
*Orthopädisch/unfallchirurgisch (551–600)*	13 (7,2 %)	70 (38,9 %)	97 (53,9 %)	180 (25,1 %)
551 Rückenschmerzen	4 (1,4 %)	10 (4,1 %)	17 (8,9 %)	31 (4,3 %)
554 Schmerzen obere Extremität	2 (0,7 %)	7 (2,9 %)	16 (8,4 %)	25 (3,5 %)
555 Schmerzen untere Extremität	6 (2,1 %)	22 (9,1 %)	14 (7,4 %)	42 (5,8 %)
556 Verletzung obere Extremität	0 (0 %)	13 (5,4 %)	29 (15,3 %)	42 (5,8 %)
557 Verletzung untere Extremität	1 (0,3 %)	18 (7,5 %)	21 (11,1 %)	40 (5,6 %)
*Respiratorisch* (651–700)	29 (10,1 %)	23 (9,5 %)	19 (10 %)	71 (9,9 %)
651 Dyspnoe	29 (10,1 %)	23 (9,5 %)	19 (10 %)	71 (9,9 %)
*Behandlungsergebnis*				
*Ambulant *(KV oder NA ambulant)	172 (59,7 %)	113 (46,9 %)	118 (62,1 %)	403 (56,1 %)
*Stationär*				
Normalstation (NC)	65 (22,6 %)	96 (39,8 %)	49 (25,8 %)	210 (29,2 %)
Intermediate-Care-Station (IMC)	42 (14,6 %)	21 (8,7 %)	21 (11,1 %)	84 (11,7 %)
Intensivstation (ICU)	9 (3,1 %)	10 (4,6 %)	2 (1,1 %)	21 (3,1 %)
*Exitus letalis*	6 (2,1 %)	3 (1,3 %)	2 (1,1 %)	11 (1,5 %)
*Mittlere Verweildauer*				
Notaufnahme, M (STD) in min	348,9 (220,6)	196,3 (95,4)	190,9 (105,6)	257,4 (177,9)
Krankenhaus, M (STD) in Tagen	9,1 (8,5)	8,7 (9,3)	7,2 (6,1)	8,5 (8,4)

*CEDIS *Canadian Emergency Department Information System, *KV* Kassenärztliche Vereinigung, *M* „mean“; *n* Patientenanzahl, *NA* Notaufnahme, *STD* Standardabweichung

Die 20 häufigsten Leitsymptome gruppierten sich in 7 Cluster (kardiovaskulär, HNO [Ohren], gastrointestinal, urogenital, neurologisch, orthopädisch/unfallchirurgisch und respiratorisch) von insgesamt 17 Symptomclustern gemäß CEDIS. Die Anzahl der Patient:innen in den Symptomclustern variierte zwischen *n* = 4 (Ohrenschmerz) und *n* = 183 (kardiovaskulär). Im gesamten Patientenkollektiv traten die Leitsymptome „Bauchschmerzen“ (14 %), „allgemeine Schwäche“ (10,4 %) und „Luftnot“ (9,9 %) am häufigsten auf. Die Prävalenz der einzelnen Leitsymptome war in den drei Modellkliniken aufgrund differenter Schwerpunkte unterschiedlich: So war das Leitsymptom „Schwäche in den Extremitäten/Symptome eines Schlaganfalls“ in der UMG, „Verletzung obere Extremität“ im EKW und „Bauchschmerzen“ das häufigste Leitsymptom im KFÜ. Lediglich das Leitsymptom „Luftnot“ trat mit vergleichbarer Häufigkeit in allen drei Modellkliniken auf (UMG 10,1 %, EKW 10,0 %, KFÜ 9,5 %).

Die durchschnittliche Verweildauer in der Notaufnahme lag im gesamten Patientenkollektiv bei 257,4 min (STD = ±177,9 min) und im Median bei 216 min. Die durchschnittliche Krankenhausverweildauer aller stationär behandelten Patient:innen lag bei 8,5 Tagen (STD = ±8,4 Tage), im Median bei 6 Tagen. Im Verlauf der notfallmedizinischen Behandlung konnten 403/718 Patient:innen (56,1 %) nach Hause entlassen werden, während 315/718 (43,9 %) Patient:innen stationär in der jeweiligen Modellklinik aufgenommen werden mussten. Von den *n* = 315 stationären Patient:innen wurden *n* = 210 (29,2 %) auf die Normalstation, *n* = 84 (11,7 %) auf die IMC Station und *n* = 21 (3,1 %) auf der ICU aufgenommen. Insgesamt sind im Beobachtungszeitraum *n* = 11 (1,5 %) Patient:innen verstorben.

### Strukturierte Ersteinschätzung mittels OPTINOFA

#### Prävalenz der OPTINOFA-Triage-Stufen

**Abb. 2:**
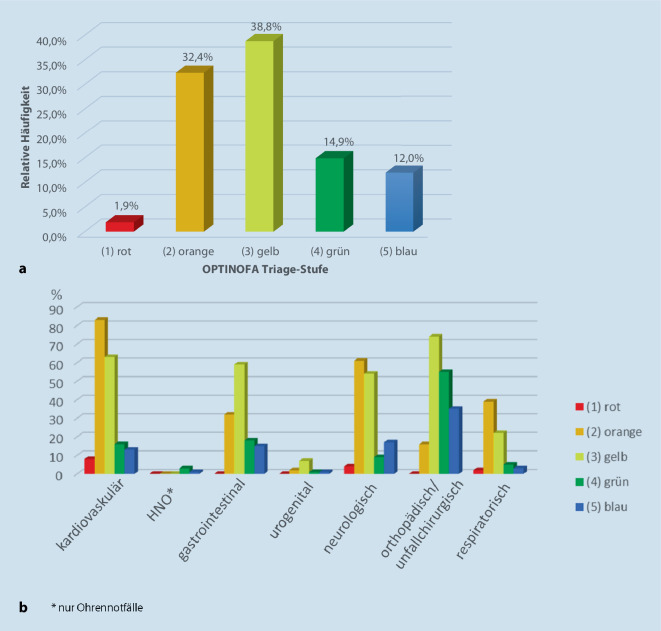
Prävalenz der OPTINOFA-Triage-Stufen im Patientenkollektiv der Pilotstudie. **a** Prozentuale Verteilung im gesamten Kollektiv. **b** Verteilung innerhalb der häufigsten Symptomcluster in der Pilotstudie

In der Pilotstudie wurden *n* = 718 Patient:innen in den drei Modellkliniken eingeschlossen und der strukturierten Ersteinschätzung mittels OPTINOFA unterzogen. Die häufigste Triagestufe, in der *n* = 279 (38,8 %) Patient:innen zugeordnet waren, war die Stufe „3 (gelb)“, gefolgt von der Stufe „2 (orange)“ (*n* = 233/32,4 %), der Stufe „4 (grün)“ (*n* = 107/14 %), der Stufe „5 (blau)“ (*n* = 85/12 %) und der Stufe „1 (rot)“ (*n* = 13/1,9 %; Abb. 2a). Die Verteilung der OPTINOFA-Triage-Kategorien in Bezug auf die häufigsten Symptomcluster ist in Abb. 2b dargestellt.

#### Zeitdauer

**Abb. 3:**
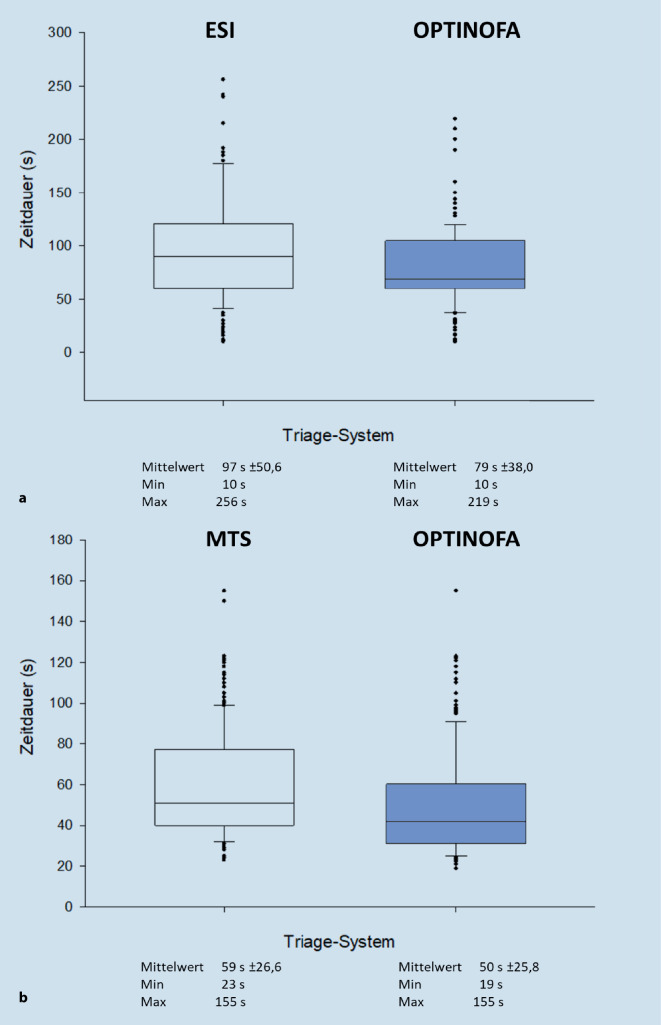
Zeitdauer der strukturierten Ersteinschätzung mittels OPTINOFA im Vergleich zu den etablierten Triage-Systemen Emergency Severity Index (*ESI*) (**a**) und Manchester Triage System (*MTS*) (**b**). *Min* Minimum, *Max* Maximum

In der Pilotstudie wurde die Zeitdauer für die Durchführung der strukturierten Ersteinschätzung mittels OPTINOFA im Vergleich zu den etablierten Triagesystemen ESI und MTS analysiert: Für die Anwendung der OPTINOFA-Triage lag die Zeitdauer in den einzelnen Modellkliniken im Mittelwert bei 90 s ± 34,4 (minimal [min] 45 s, [max] 200 s; UMG), 68 s ± 38,7 (min 10 s, max 210 s; KFÜ) bzw. 50 s ± 25,8 (min 19 s, max 155 s, EKW). Hierbei ergab sich kein Unterschied zu den etablierten Triagesytemen ESI (Abb. 3a) bzw. MTS (Abb. 3b).

### Validierung

#### Vergleich der OPTINOFA-Triage-Stufe mit der tatsächlichen Versorgungsstufe

**Abb. 4:**
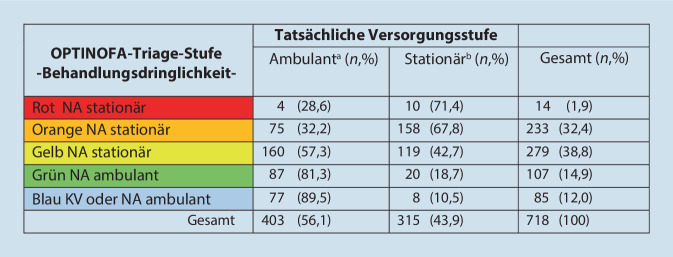
Analyse der OPTINOFA-Triage-Stufe (Behandlungsdringlichkeit) in Bezug zur tatsächlichen Versorgungsstufe. ^a^Weiterleitung in die KV oder Entlassung; ^b^Stationäre Aufnahme auf die Normalstation (NC), Intermediate-Care(IMC)-Station oder Intensivstation (ICU); *KV* kassenärztliche Versorgung, *NA* Notaufnahme

Zur Validierung der strukturierten Ersteinschätzung mittels OPTINOFA wurden für alle eingeschlossenen Notfälle zunächst die zugewiesene OPTINOFA-Triage-Stufe in Bezug auf die Behandlungsdringlichkeit (a) und die festgelegte Versorgungsstufe (b) mit der tatsächlichen Versorgungsstufe verglichen (Tab. 3 und Abb. 4). Hierbei ergab sich für die OPTINOFA-Triage eine Sensitivität von 91,1 % und eine Spezifität von 40,7 %.

**Tab. 3:** Analyse der OPTINOFA-Triage-Stufe (Versorgungsstufe ambulant/stationär) in Bezug zur tatsächlichen Versorgungsstufe; Sensitivität: 287/315 = *91,1* *%; *Spezifität: 164/403 = *40,7* *%*

OPTINOFA-Triage-Stufe Versorgungsstufe	Tatsächliche Versorgungsstufe		
	Ambulant (*n*, %)	Stationär (*n*, %)	Gesamt (*n*, %)
Ambulant	164 (40,7; 85,4)	28 (9; 14,6)	192 (26,8)
Stationär	239 (59,3;45,4)	287 (91; 54,6)	526 (73,2)
Gesamt	403 (56,1)	315 (43,9)	718 (100)

**Abb. 5:**
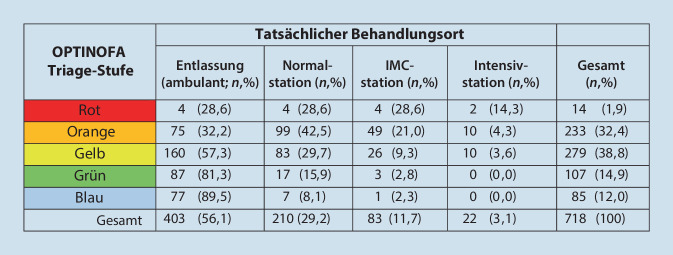
Analyse der Korrelation zwischen OPTINOFA Triage-Stufen und tatsächlichem Behandlungsort. *IMC* Intermediate Care

#### Analyse der Korrelation zwischen OPTINOFA-Triage-Stufe und tatsächlichem Behandlungsort

Die OPTINOFA-Triage-Stufen korrelierten hoch signifikant mit dem Behandlungsort stationär (*p* < 0,001). Insgesamt wurden in der Pilotstudie 315/718 (43,9 %) Patient:innen stationär aufgenommen. Davon haben Patient:innen mit den hohen Dringlichkeitsstufen (1 [rot] und 2 [orange]) eine signifikant höhere Hospitalisierungsrate mit stationärer Aufnahme auf einer Normal‑, IMC-Station oder ICU. Demgegenüber repräsentierten die Notfälle mit den OPTINOFA-Triage-Stufen 3 (gelb), 4 (grün) und 5 (blau) den überwiegenden Anteil an Patient:innen, die entlassen und damit ambulant versorgt wurden (Abb. 5).

**Abb. 6:**
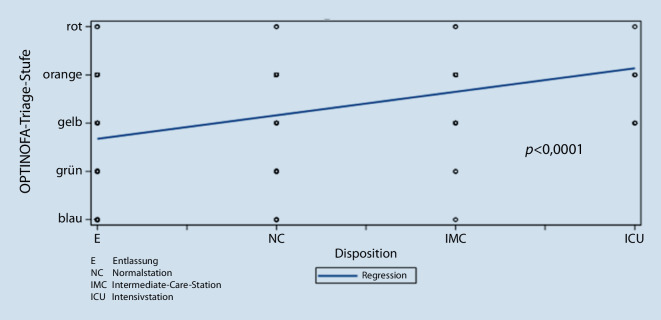
Korrelation der OPTINOFA-Triage-Stufen mit der Disposition (Behandlungsort)

In der Korrelationsanalyse zwischen OPTINOFA-Triage-Stufe und tatsächlichem Behandlungsort ergab sich daher eine signifikante Korrelation (Spearman’s Rank Correlation *p* < 0,0001; Abb. 6).

#### Prädiktion des Vorhersagewerts der OPTINOFA-Triage-Stufe in Bezug auf den definitiven Behandlungsort

**Abb. 7:**
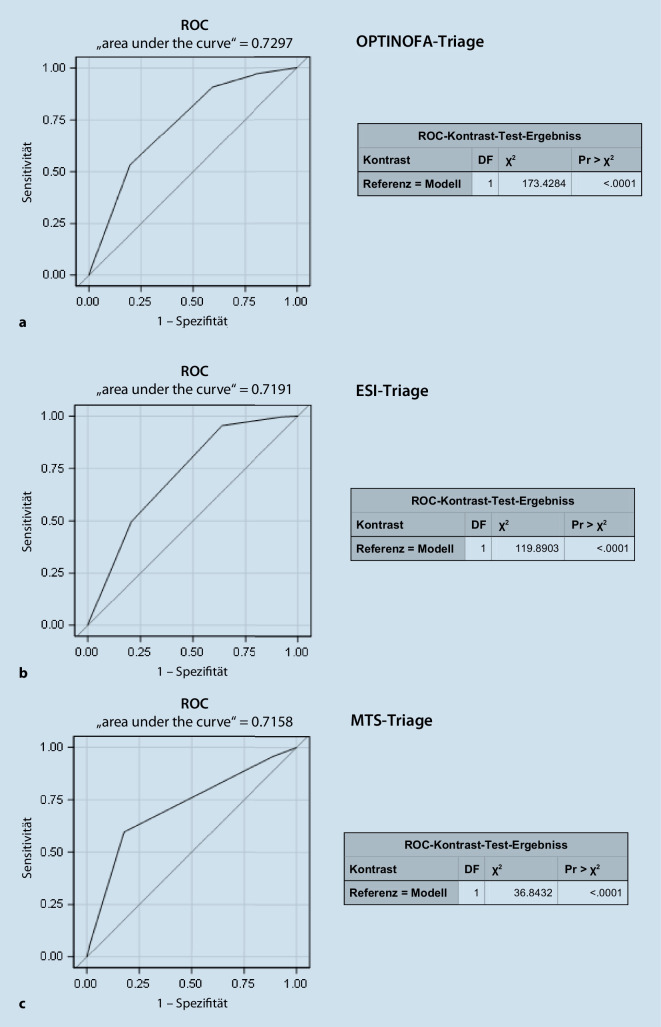
Receiver-operating-characteristic(*ROC*)-Analyse der Triagestufen gemäß OPTINOFA (**a**), Emergency Severity Index (*ESI*; **b**) oder Manchester Triage System (*MTS*; **c**) und der Disposition in Bezug auf den definitiven Behandlungsort

Mit der ROC-Analyse wurde die Fähigkeit des OPTINOFA-Triage-Systems zur Vorhersage der Disposition in Bezug auf den definitiven Behandlungsort (ambulant vs. stationäre Behandlung auf der Normal‑, IMC-Station oder ICU) untersucht. Die Ergebnisse zeigen, dass die Fläche unter der Kurve bei 0,7297 lag (χ^2^ = 173,4284, DF = 1; *p* < 0,0001; Abb. 7a).

In der parallelen Analyse ergab sich für das Triagesystem ESI (Abb. 7b) eine AUC von 0,7191 (χ^2^ 119,89, *p* < 0,001) und für MTS (Abb. 7c) eine AUC von 0,7158 (χ^2^ 36,84; *p* < 0,001). Somit wurde im Rahmen dieser Pilotstudie im Vergleich mit den etablierten Triagesystemen ESI und MTS mit Durchführung der OPTINOFA-Triage die beste AUC und damit die beste Vorhersage der Disposition in Bezug auf den tatsächlichen Behandlungsort erzielt. Die OPTINOFA-Triage war damit zur Vorhersage des tatsächlichen Behandlungsortes mindestens genauso gut wie die etablierten Triagesysteme geeignet. Es wurde aber kein Test zum Vergleich der Systeme untereinander bezüglich ihrer AUC, sondern ein Vergleich der einzelnen Systeme mit Zufallsentscheidungen durchgeführt. Aufgrund der ähnlichen Werte zwischen den Systemen wäre auch kein signifikanter Unterschied zu erwarten.

#### Assoziation zwischen OPTINOFA-Triage-Stufe und Verweildauer im Krankenhaus bzw. in der Notaufnahme

**Abb. 8:**
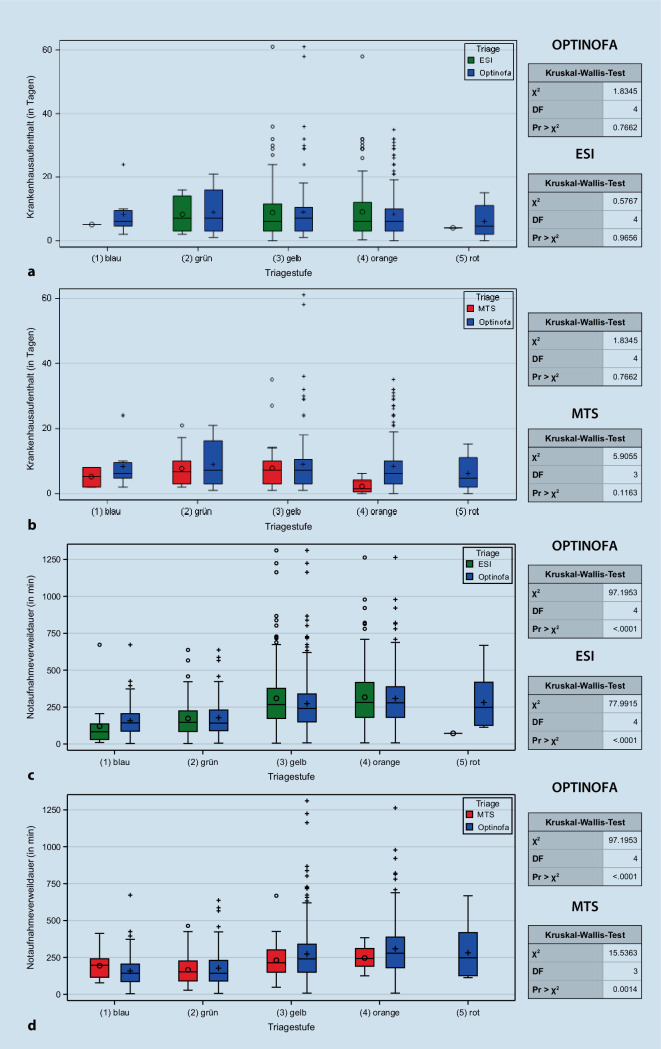
Analyse der Assoziation zwischen OPTINOFA-Triage-Stufe und Krankenhausverweildauer (**a**, **b**) sowie der Verweildauer in der Notaufnahme (**c**, **d**) im Vergleich zum Emergency Severity Index (*ESI*) und Manchester Triage System (MTS)

In der Pilotstudie wurde die stationäre Verweildauer im Krankenhaus für alle stationär aufgenommenen Notfälle erhoben und die Assoziation mit den primär zugewiesenen Triagestufen analysiert. In der Boxplotdarstellung (Abb. 8) zeigte sich dabei weder für die OPTINOFA-Triage noch für die ESI-Triage-Stufen (Abb. 8a) oder die MTS-Triage-Stufen (Abb. 8b) eine relevante Assoziation. Eine Prädiktion der Krankenhausverweildauer war daher mit keinem der eingesetzten Triagesysteme möglich. Im Kruskal-Wallis-Test ergab sich daher für alle drei Triagesysteme kein signifikanter Zusammenhang zwischen der Triagestufe und der stationären Krankenhausverweildauer.

Demgegenüber fand sich sowohl für OPTINOFA als auch für ESI und MTS ein signifikanter Zusammenhang zwischen der Triagestufe und der Verweildauer in der Notaufnahme (Abb. 8c, d); die Signifikanzniveaus lagen für OPTINOFA und ESI bei *p* < 0,0001, für MTS bei *p* = 0,0014. Hierbei lag die mittlere Verweildauer für die dringlicheren Notfälle (1 [rot], 2 [orange]) in der Regel höher als die Verweildauer in den niedrigeren Dringlichkeitskategorien (4 [grün], 5 [blau]).

#### Analyse der Korrelation zwischen der OPTINOFA-Triage-Stufe und der Mortalität

**Abb. 9:**
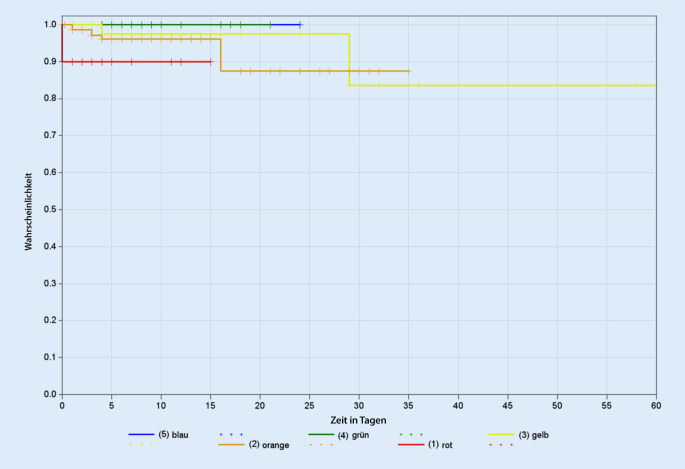
Kaplan-Meier-Kurven zur Darstellung des Mortalitätsrisikos in Abhängigkeit von der OPTINOFA-Triage-Stufe

Die Überlebenswahrscheinlichkeit von Patient.innen mit den nichtdringlichen OPTINOFA-Stufen 4 (grün) und 5 (blau) lag in der Pilotstudie bei 100 %. Demgegenüber ergab sich in den Kaplan-Meier-Kurven für Patient:innen mit den OPTINOFA-Stufen 1 (rot), 2 (orange) oder 3 (gelb) wie erwartet ein höheres Mortalitätsrisiko. Der Zusammenhang zwischen den OPTINOFA-Triage-Stufen und der Überlebenswahrscheinlichkeit ist in Abb. 9 dargestellt; die korrespondierenden Analysen für die ESI- bzw. MTS-Triage finden sich im Supplement (Abb. S1, S2).

## Diskussion

In Deutschland bestehen gemäß Gutachten des Sachverständigenrats für Gesundheit erhebliche Defizite im Status quo der Notfallversorgung [14]. Hierbei führen u. a. die hohen Fallzahlen in den ZNA der Krankenhäuser zu rezidivierend auftretenden Overcrowding-Szenarien. Eine wesentliche Ursache für diese Problematik besteht in der Schwierigkeit einer zuverlässigen präklinischen Steuerung der Patientenströme in der Notfallversorgung. In den ZNA der Krankenhäuser erfolgt eine stringente Steuerung mithilfe von Triagesystemen, die die Notfallpatient:innen nach Behandlungsdringlichkeit identifizieren und kategorisieren. Für den deutschsprachigen Raum haben sich ESI und MTS als 5‑stufige Systeme durchgesetzt [11].

Die bisherigen Triageinstrumente sind jedoch ausschließlich für die medizinische Ersteinschätzung hinsichtlich der Behandlungsdriglichkeit, also Wartezeit bis Arztkontakt, in der Notaufnahme validiert. Die Zuordnung einer Versorgungsstufe im Sinne einer ambulanten oder stationären Versorgung bleibt bisher unberücksichtigt. Um eine ressourcenspezifische Zuordnung bereits zum Zeitpunkt der medizinischen Erstsichtung zu ermöglichen und Patient:innen der im Gesundheitssystem vorgesehenen Versorgungsstruktur zuzuweisen, wird in einer aktuellen Richtlinie des GVWG ein validiertes Instrument zur strukturierten Ersteinschätzung gefordert, das sowohl die Behandlungsdringlichkeit als auch den Behandlungsort sicher zuweist und digital dokumentiert wird [10].

Welches System hierfür geeignet sein könnte, wurde bereits seit einigen Jahren in verschiedenen Studien untersucht. In zwei Studien wurde versucht, das bereits zur Einschätzung der Behandlungsdringlichkeit angewendete MTS-Triage-System auch zur Einschätzung des notwendigen Behandlungsortes zu verwenden. [17, 23]. Beide Studien kamen zu dem Schluss, dass MTS in der existierenden Form nicht dazu geeignet ist, Notfallpatient:innen in die ambulante Versorgungsebene ohne Risiko weiterzuleiten. Ebenso wurde in einer weiteren Studie zur Bewertung der ESI-Triage im Hinblick auf Zuteilung der Versorgungsebene bei Erstsichtung festgestellt, dass ESI zur Steuerung von Notfallpatient:innen in Versorgungsstrukturen außerhalb der Notaufnahme nicht geeignet ist [21]. Darüber hinaus ist das im vertragsärztlichen Bereich eingesetzte Instrument zur Telefontriage, das „strukturierte medizinische Ersteinschätzung in Deutschland (SmED)“, bisher nicht für die Anwendung in Kliniken validiert und die Daten der bisherigen Studien dazu sind nicht veröffentlicht [24].

Im Innovationsfondsprojekt OPTINOFA wurde demgegenüber ein neues, digitales Triageinstrument entwickelt, das erstmalig eine strukturierte Ersteinschätzung der Behandlungsdringlichkeit und zeitgleich der Versorgungsstufe ermöglicht. Zur Validierung des Systems auf Basis der vom G‑BA geforderten Gütekriterien gemäß § 3 der Richtlinie [9], d. h. Diskriminationsfähigkeit, Vollständigkeit, Objektivität, Reliabilität und Validität, wurde die vorliegende Pilotstudie durchgeführt, in die eine weitgehend repräsentative Population von Notfallpatient:innen in 3 Modellkliniken eingeschlossen wurde.

Das Triageinstrument OPTINOFA ist ein 5‑stufiges Triagesystem, das als intelligenter Assistenzdienst auf mobilen Endgeräten zu Verfügung gestellt und digital dokumentiert wird. Die Anwendung erfolgte durch pflegerisches oder ärztliches Personal mit Kenntnissen in der Notfallmedizin, sodass die vom G‑BA geforderte Objektivität und Reliabilität erfüllt sind. Ebenso handelt es sich um ein Instrument, das vergleichbar mit ESI und MTS innerhalb einer Zeitdauer von etwa 60 s durchgeführt werden kann, wie die Ergebnisse der Pilotstudie in allen 3 Modellkliniken zeigen.

Zur Bewertung der Validität eines Triageinstruments gibt es im Hinblick auf die Bestimmung der Behandlungsdringlichkeit keinen Goldstandard. Daher bedient man sich mithilfe von Surrogatparametern der Evaluation der sog. Konstruktvalidität [26]. Die Krankenhausmortalität als einer dieser Surrogatparameter wird auch zur Bewertung der Sicherheit einer Triageskala verwendet. Sie sollte in der niedrigsten Dringlichkeitsstufe im Idealfall 0 % betragen [7]. Dies wurde in der vorliegenden Pilotstudie für die Anwendung von OPTINOFA in allen 3 Kliniken sogar für die OPTINOFA-Triage-Stufen 4 und 5 erfüllt.

Zur Bestimmung der Behandlungsdringlichkeit wurden neben der Mortalitätsrate in dieser Pilotstudie auch die klinischen Verläufe aller Fälle retrospektiv analysiert und die stationären Aufenthalte auf IMC-, Intensiv- oder Überwachungsstationen, Komplikationen sowie sofort durchgeführte Operationen und Interventionen als Marker für eine „hohe Behandlungsdringlichkeit“ bei Aufnahme bewertet [27]. Die Ergebnisse der Pilotstudie zeigen, dass OPTINOFA in mehr als der Hälfte der Fälle richtig mit der Einschätzung eines Patienten als „hoch dringlich“ lag und zwar mit einer Sensitivität von 91,1 % und einer signifikanten Korrelation der OPTINOFA-Behandlungsstufe zum Behandlungsort stationär. Darüber hinaus wurde mittels OPTINOFA auch die strukturierte Ersteinschätzung von ambulant behandelbaren Notfallpatient:innen mit möglicher Weiterleitung in den vertragsärztlichen Sektor mit mittlerer Sensitivität, aber hoher Spezifität richtig erkannt; fast die Hälfte der ambulant behandelten Notfallpatient:innen wurde mit OPTINOFA in die Stufe 4 und 5 eingeschätzt. Somit wäre die im § 3 geforderte Diskriminationsfähigkeit des Triagetools im Fall von OPTINOFA erfüllt.

Die niedrige Spezifität von OPTINOFA (40,7 %) bei der Einstufung der Behandlungsdringlichkeit deckt sich mit den Ergebnissen der Studien zur Validierung anderer Triageinstrumente wie ESI oder MTS [27]. Dies zeigte sich in der vorliegenden Studie mit Anwendung von OPTINOFA in den 3 Modellkliniken auch bei der Disposition der Patient:innen in die stationäre bzw. ambulante Behandlung. Erklärbar ist dies zum Teil mit unterschiedlichen Vorgaben bezüglich der Weiterleitungen in die vertragsärztliche Versorgung bzw. ambulanten Behandlungen innerhalb der ZNA der Modellkliniken, den individuellen Risikofaktoren der Notfallpatient:innen und den unterschiedlichen Ressourcen, die für das ambulante Setting vorgehalten werden.

Nichtsdestotrotz muss man bei der gegenwärtigen Diskussion zur Reform der Notfallversorgung durch den G‑BA v. a. die Bedeutung der niedrigen Spezifität im Blick haben: In OPTINOFA-Stufe 5 (blau) wurden 9,4 % der eigentlich als entlassfähig eingestuften Patient:innen stationär behandelt, in Stufe 4 sogar 18,7 %. Somit wurden 14,6 % der grün bzw. blau triagierten Patienten stationär aufgenommen, was sich mit Ergebnissen aus Studien anderer Triagesysteme deckt [21]. Zwischen 3 und 45 % der initial in die niedrigsten Dringlichkeitskategorien eingestuften Patienten [15] und auch 10 % der nichtdringlich eingestuften „Selbstzuweiser“ bedurften stationärer Aufnahmen [23]. Natürlich kann die Entscheidung zwischen ambulant und stationär vielfältige und nicht immer rein medizinische Gründe haben. Allerdings ist die Abklärung eines stationären Behandlungsbedarfs mit Mitteln einer Notaufnahme und dem Resultat einer abschließenden ambulanten Behandlung systemimmanent und setzt eben regelhaft einen Diagnostikprozess voraus [16]. Immerhin mussten 1,2 % der blau (OPTINOFA-Stufe 5) triagierten Patient:innen auf einer IMC-Station behandelt werden und sogar 2,8 % der Patienten in Stufe 4. In Anbetracht der Diskussion um die (Nicht‑)Vergütung ambulanter Behandlungen in Kliniken und die verpflichtende Weiterleitung von „nichtdringlich“ triagierten Patient:innen muss auch überlegt werden, welche „Fehlerquote eines Triagesystems“ dann vertretbar ist; d. h. welche Risiken bestehen für diese 1,2 % der schlussendlich IMC-pflichtigen Patient:innen bei einer Weiterleitung in den ambulanten Sektor. Dieses Faktum unterstreicht, dass es auch für zukünftige Validierungsstudien schwierig sein wird, Grenzwerte hinsichtlich der Diskriminationsfähigkeit zu empfehlen, die sowohl den ökonomischen wie den ethischen Anforderungen einer größtmöglichen Patientensicherheit Rechnung tragen. Erschwerend kommt hinzu, dass jede Studie in der Frage der Diskriminierung zwischen den Sektoren sowohl die Tageszeit als auch den Wochentag sowie die potenziell zur Verfügung stehenden Ressourcen der Notdienstpraxen berücksichtigen muss. Eine Weiterleitung an Notdienstpraxen in oder am jeweiligen Krankenhaus, wie sie im europäischen Ausland bereits praktiziert wird [20], könnte die Lösung sein, einerseits die Patientensicherheit zu gewährleisten und andererseits die ZNA zu entlasten und Overcrowding-Situationen zu vermeiden.

## Limitationen

Die Studie wurde in drei Modellkliniken unterschiedlicher Versorgungsstufen in Deutschland durchgeführt. Inwieweit die Ergebnisse auf andere internationale Gesundheitssysteme übertragbar sind, sollte in weiteren Studien untersucht werden.

In Bezug auf die Auswertungen zur Krankenhausmortalität wurden im Rahmen dieser Pilotstudie nur die Zeiträume bis zur Entlassung aus dem Krankenhaus betrachtet. Todesfälle, die unmittelbar nach dem stationären Aufenthalt im häuslichen Umfeld oder einem anderen Krankenhaus aufgetreten sein könnten, sind in die Evaluation nicht eingeflossen.

Zur Validierung des neuen Triagesystems OPTINOFA wurde im Rahmen des Innovationsfondsprojekts zunächst eine Pilotstudie durchgeführt. In diese Studie wurden in einem kurzen Beobachtungszeitraum *n* = 718 Notfälle, jedoch mit einem weitgehend repräsentativen Patientenkollektiv (vgl. Tab. 1 und Abb. 1), eingeschlossen. Aufgrund der geringen Fallzahlen bleiben die Ergebnisse der nachfolgend durchgeführten bundesweiten clusterrandomisierten und kontrollierten Interventionsstudie abzuwarten.

Darüber hinaus ist die im § 3 der G‑BA-Richtlinie zur Ersteinschätzung geforderte Vollständigkeit des Ersteinschätzungsverfahren bisher noch nicht gegeben, da das OPTINOFA-Triage-System bislang nur für die 20 häufigsten CEDIS-Leitsymptome entwickelt wurde.

## Ausblick

Zur Bewältigung der Herausforderungen in den ZNA in Deutschland ist zukünftig eine enge Kooperation zwischen den beiden Sektoren der ambulanten und stationären Notfallversorgung unerlässlich. Unter dem Primat der Patientensicherheit bedarf es dabei einer gezielten Steuerung der Patientenströme in diese Sektoren, um eine bedarfsgerechte Notfallversorgung sicherzustellen und Über‑, Unter- und Fehlversorgung zu vermeiden. Mit der Entwicklung und Validierung eines neuen Triageinstruments zur strukturierten Ersteinschätzung der Behandlungsdringlichkeit und gleichzeitig Festlegung des Versorgungssektors kann diese Problematik erfolgreich adressiert werden. Dies setzt die Durchführung klinischer Studien voraus, die die Kriterien Kontrolle, Randomisierung und multizentrisches Design erfüllen.

## Fazit für die Praxis

- Im Innovationsfondsprojekt „Optimierung der Notfallversorgung durch strukturierte Ersteinschätzung mittels intelligenter Assistenzdienste“ (OPTINOFA) wurde ein sicheres und valides Instrument zur strukturierten Ersteinschätzung von Notfällen in der Zentralen Notaufnahme entwickelt, das die zeitgleiche Bestimmung der Behandlungsdringlichkeit und erforderlichen Versorgungsstufe erlaubt.
- Die Zeitdauer der Anwendung der OPTINOFA-Triage liegt innerhalb von maximal 3 min und ist damit vergleichbar mit den etablierten Instrumenten Emergency Severity Index (ESI) und Manchester Triage System (MTS).
- In Bezug auf die Prädiktion des definitiven Behandlungsorts (Disposition) ist die OPTINOFA-Triage den etablierten Triagesystemen überlegen. Es besteht darüber hinaus eine signifikante Assoziation zwischen der OPTINOFA-Triage-Stufe und der Verweildauer in der Notaufnahme.
- Die OPTINOFA-Triage erfüllt damit die Gütekriterien der Diskriminationsfähigkeit, Reliabilität, Objektivität und Validität für Triagesysteme.
- Durch eine transsektorale Steuerung der Patientenströme eröffnet das innovative Triageinstrument OPTINOFA neue Perspektiven für die Entlastung der Notaufnahmen und eine bedarfsgerechte Notfallversorgung in Deutschland.

## Supplementary Information

Abb. S1 Kaplan-Meier-Kurven zur Darstellung des Mortalitätsrisikos in Abhängigkeit von der ESI-Triage-Stufe

Abb. S2 Kaplan-Meier-Kurven zur Darstellung des Mortalitätsrisikos in Abhängigkeit von der MTS-Triage-Stufe
