# Supplementary material for: OPTINOFA—Intelligent assistance service for structured assessment in the emergency department
Source: Med Klin Intensivmed Notfmed. 2024 Mar 27;120(3):208–21. [Article in German] doi: 10.1007/s00063-024-01126-y (PMC11961501; doi:10.1007/s00063-024-01126-y)
Supplement: Supplementary file 1 — Abb. S1 Kaplan-Meier-Kurven zur Darstellung des Mortalitätsrisikos in Abhängigkeit von der ESI-Triage-Stufe [file 63_2024_1126_MOESM1_ESM.docx]

**Abb. S1 Kaplan-Meier Kurven zur Darstellung des Mortalitätsrisikos in Abhängigkeit von der ESI Triage-Stufe**

**
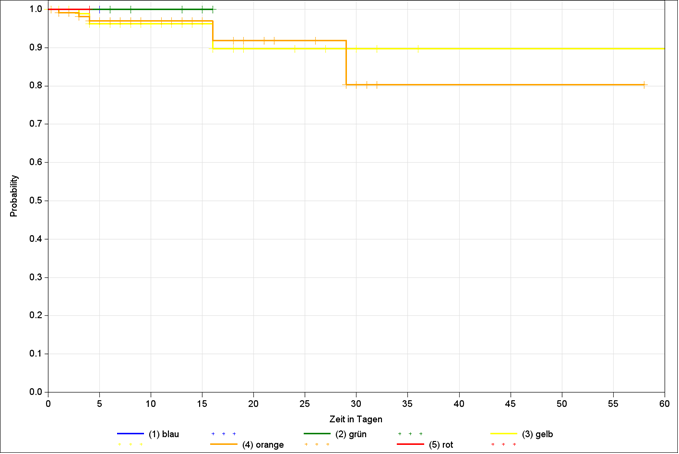
**
